# Effects of exercise intervention on balance function in children with cerebral palsy: a systematic review and meta-analysis of randomized controlled trials

**DOI:** 10.1186/s13102-024-00922-5

**Published:** 2024-08-07

**Authors:** Junjian Xiao, Linghong Liu, Nan Tang, Chao Yi

**Affiliations:** 1 University of Health and Rehabilitation Sciences, Qingdao, China; 2 Institute of Physical Education, Jiangsu Normal University, Xuzhou, China; 3 Sports Department, Sanjiang University, Nanjing, China; 4 School of Sports Science, Qufu Normal University, Qufu, China

**Keywords:** Children, Cerebral palsy, Exercise, Meta-analysis, Balance

## Abstract

**Objective:**

To determine the effectiveness of exercise intervention on postural balance, gait parameters, and muscle strength in children with cerebral palsy by quantifying the information from randomized controlled trials (RCTs).

**Methods:**

We conducted a systematical search for RCTs from the databases, including PubMed, ISI Web of Science, and Scopus using a between-group design involving children with cerebral palsy and assessing the effect of exercise intervention on postural balance, gait parameters, and muscle strength. The specified inclusion criteria were determined by the PICOS tool. The outcomes of included studies were evaluated by meta-analysis, and subgroup and sensitivity analyses were conducted to analyze the observed heterogeneities using Review Manager 5.4 and Stata version 18.0. The revised Cochrane risk of bias tool for randomized trials (RoB 2) was used to evaluate the risk of bias and quality of the included studies.

**Results:**

Twenty-four studies were included in this meta-analysis, with 579 children with cerebral palsy. Exercise intervention showed a statistically significant favorable effect on gross motor function (SMD = 0.32; 95%CI [0.03 to 0.61]; *I*^2^ = 16%), anteroposterior stability index (SMD = -0.93; 95%CI [-1.69 to -0.18]; *I*^2^ = 80%), and mediolateral stability index (SMD = -0.60; 95%CI [-1.16 to -0.03]; *I*^2^ = 73%) compared to control group among children with cerebral palsy. None of the above meta-analyses exhibited publication bias, as indicated by Egger’s test with p-values greater than 0.05 for all.

**Conclusions:**

Exercise is effective in improving gross motor function and balance in children with cerebral palsy. Due to the lack of studies examining the efficacy of each exercise type, we are unable to provide definitive training recommendations.

**Supplementary Information:**

The online version contains supplementary material available at 10.1186/s13102-024-00922-5.

## Introduction

Cerebral palsy (CP) refers to a group of permanent disorders affecting the development of movement and posture, leading to limitations in activity. These disorders are attributed to non-progressive disturbances in the developing fetal or infant brain [1]. CP frequently presents with motor problems, commonly accompanied by sensory, cognitive, communication, perceptual, and/or behavioral disorders [2–4]. Additionally, individuals with CP may also experience seizures, nutritional issues, dysphagia, and oral motor dysfunction, resulting in a subsequent decrease in physical activity and overall quality of life [5–7]. Consequently, individuals with CP depend on a multitude of health and educational services [8, 9]. The Gross Motor Function Classification System (GMFCS) is a functional classification system that categorizes children and youth with CP based on their current gross motor skills and limits, as well as their requirement for assistive technology [10]. GMFCS I often exhibit unrestricted ambulation, however may encounter certain limitations in more demanding circumstances. GMFCS V typically have significant mobility impairments, often necessitating the use of assistive equipment to facilitate movement. Neural plasticity makes the early years crucial for children’s motor function development [11].

The rehabilitation of balance and walking ability is crucial for children diagnosed with cerebral palsy [12, 13]. Balance refers to the capacity to sustain an upright position or vertical alignment while engaging in various activities, including sitting, standing, and walking [14, 15]. It is contingent upon the intricate interplay of the central nervous system, musculature, physical power, proprioception, body alignment, visual perception, and the vestibular system [16–18]. The significance of early intervention in optimizing long-term functionality in children with CP and their prospects for leading a fulfilling life has been extensively recognized [19, 20]. The progression of intervention has shifted the focus from primarily addressing the underlying symptoms and impairments to improve function, to instead focusing on training activities and real-life tasks that hold significance to the individual [12]. In addition, there is a requirement to promote increased engagement in exercise, in line with recommendations, to attain elevated levels of fitness, diminish risk factors for diseases, and minimize subsequent problems such as premature functional decline [21]. Exercise programs for cerebral palsy exhibit significant variation in terms of their types, such as gait training, body-weight–supported treadmill training, balance training, or multi-component approaches, and the efficacy of different exercises has not been established in improving the functional abilities of children with cerebral palsy [13, 22–24].

Currently, several systematic reviews and meta-analyses have demonstrated the potential impact of exercise interventions on children with CP. A meta-analysis by Liang et al. has illustrated that exercise interventions have a significant effect on higher levels of gait speed and muscle strength and no impact on gross motor function, including 834 children with CP for quantitative analysis [25]. Another meta-analysis involving 847 children and adolescents with spastic CP from 27 studies has shown positive effects on muscle strength, balance, gait speed, or gross motor function in strength training programs [26]. Nevertheless, despite being administered appropriately, strength training did not demonstrate efficacy in enhancing gait speed in a meta-analysis of rehabilitation interventions among children with CP [27]. And aerobic exercise was not effective for muscle strength, spasticity, gait parameters, and quality of life in aerobic exercise meta-analysis on the functioning and quality of life of children and adolescents with CP [28]. However, results regarding balance are rarely reported. Existing evidence suggests that balance plays a crucial role in the capacity to walk independently and is a substantial predictor of gait function [14]. Therefore, it is important to clarify the effectiveness of exercise interventions for balance function in children with CP.

Additionally, the application of meta-analysis can help address and close several important knowledge gaps by: (1) offering solid proof of the impact of exercise intervention on functional balance abilities in children with cerebral palsy and (2) offering suggestions for the efficacy of exercise regimens as protocols to boost postural balance, gait parameters, and muscle strength.

Therefore, the objective of this study was to conduct a systematic review and meta-analysis of RCTs to evaluate the effect of different exercises on cerebral palsy in children. This knowledge is crucial for guiding clinical practice for the exercise intervention of children with cerebral palsy.

## Methods

This study was conducted following the Preferred Reporting Items for Systematic Reviews and Meta-Analyses (PRISMA) guidelines [29] and has been registered in the PROSPERO database with the ID: CRD42024501866.

### Search strategy

A systematic search was conducted in PubMed, ISI Web of Science, and Scopus databases using the following search terms: “cerebral palsy” AND “children” AND “exercise” AND “balance” from their inception date to Jan 31, 2024. The specifics regarding the search terms and processes are shown in supplementary Table 1. Two reviewers (JJX and LHL) evaluated the identified publications separately to determine their eligibility. Any conflicts were handled by a third reviewer (NT).

### Selection criteria

The studies were eligible for inclusion based on the specified inclusion criteria by PICOS tool [30]: (P) population: participants were children under the age of 18 years with CP; (I) intervention: the included studies were any type of exercise intervention; (C) Comparator: the potential comparators for this study included a control group with conventional therapy; (O) outcomes: postural balance, gait parameters, and muscle strength were outcomes of this study; (S) study type: the included studies were all RCTs trials.

Publications that met the following criteria were not included: (1) The following were excluded: reviews, comments, letters, animal studies, protocols, conference papers, and case reports. (2) The study compared two different exercise interventions. (3) Studies with inadequate data or unavailable statistical analysis were excluded. (4) Cross-over trials were excluded.

Two reviewers (JJX and LHL) autonomously completed the initial screening of titles and abstracts, then thoroughly examined full-text publications using EndNote X9 (Clarivate Analytics, Philadelphia, PA, USA). Discrepancies were resolved by seeking input from a third reviewer (NT).

### Data extraction

Two separate authors (NT and JJX) with the check of a third reviewer (LHL) extracted the following data from the included studies: author, the year of publication, the study design, participants, age, sex, weight, height, BMI, sample size, the training protocol, classification of cerebral palsy, and outcomes (postural balance, gait parameters, and muscle strength).

### Risk of bias assessment

The Cochrane Handbook version 5.1.0 tool [31] was used to evaluate the risk of bias (ROB) for primary outcomes in RCTs by two reviewers (JJX and LHL), including 7 domains: randomized sequence generation, treatment allocation concealment, blinding of participants, personnel, incomplete outcome data, selective reporting, and other sources of bias. Each item can be evaluated as low, unclear, or high risk of bias based on the RCTs by RevMan 5.4. Discrepancies were discussed and resolved by seeking a third reviewer (NT).

### Data synthesis and statistical analysis

The effect size of the control and intervention groups with exercise intervention was calculated using the standard mean differences (SMD) and standard deviation (SD) of outcome variables. To determine the SMD and its SD in the control and intervention groups, we used the following formula: (1) mean = mean _post_-mean _baseline_, (2) SD = square root ((SD_baseline_)^2^ + (SD_post_)^2^) − (2r × SD_baseline_ × SD_post_)), *r* = 0.5.

The statistical analysis was performed using the Cochrane Review Manager 5.4. The SMD, SD, and number of participants in the experimental and control groups were entered into RevMan 5.4 to calculate the effect size in the completed experiments. Heterogeneity among studies was evaluated by using I^2^ statistics in all analyses. The random-effects model was utilized when the I^2^ statistic exceeded 50%. Alternatively, the fixed-effects model was employed.

Publication bias was identified through the use of a funnel plot. Afterward, Egger regression was utilized to evaluate the asymmetry of the funnel plot utilizing Stata version 18.0. A p-value less than 0.05 indicated the lack of publishing bias. Sensitivity analyses were conducted to assess the reliability of the overall estimate by excluding individual studies using Stata version 18.0.

## Results

### Study identification

**Fig. 1:**
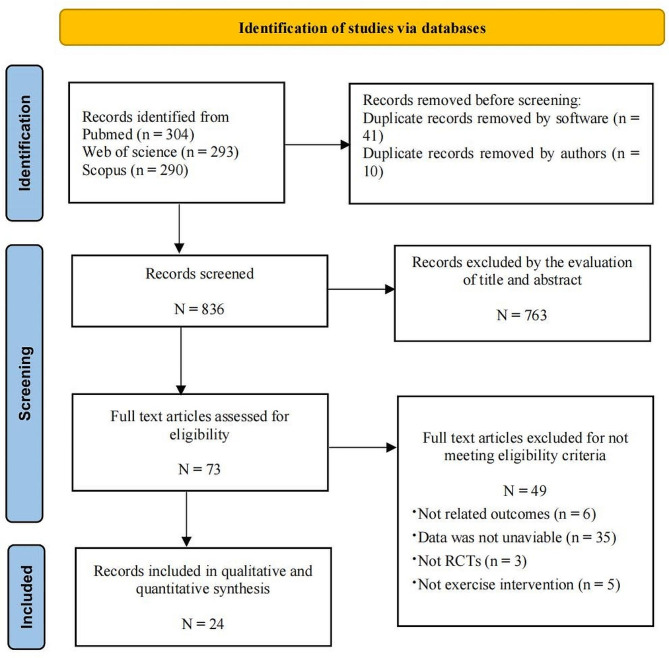
Flow diagram of studies search and screen

We conducted a search across three databases, resulting in the identification of 887 publications. Afterward, we evaluated them, selecting 24 papers [32–55] for systematic review and meta-analysis (Fig. 1 and Supplementary Table 1).

### Study characteristics

This meta-analysis included 579 children (age range: 2.5–16.0 years) with cerebral palsy from 24 studies (Exercise intervention, *n* = 294; Control, *n* = 285). Gross motor function measure (GMFM) [33, 36, 37, 43, 46, 49, 53, 55], mobility [33, 43, 46–49, 52, 55] and postural balance [32, 37, 38, 41–43, 47, 50] were assessed in 8 studies, and gait speed [32, 36, 48, 53] and muscle strength [33, 38, 46, 48] were assessed in 4 studies. The research was conducted in multiple countries worldwide: Egypt [32, 35, 38, 39], New Zealand [33], Turkey [34, 46, 47, 52, 55], Greece [36], Denmark [37], Chile [40], Spain [41], Brazil [42, 43], India [44, 50], South Africa [45], Thailand [48], USA [49, 51], and France [53, 54]. The main characteristics and exercise protocol of the included studies are shown in Table 1 and Supplementary Table 2.

**Table 1:** Main characteristics of included RCTs

Author	Region	Sex	Age (Year)	Weight (kg)	Height (cm)	BMI (kg/m^2^)	*N* Experimental group	*N* Control group	Duration	Exercise
Abd, et al. (2014)	Egypt	M (*n* = 13) F (*n* = 17)	8.8	26.75	121.3	18.18	15	15	8-week	Postural balance training
Adaikina, et al. (2023)	New Zealand	M (*n* = 4) F (*n* = 5)	2.5–4.8	0.26 (z-score)	−0.28 (z-score)	0.63 (z-score)	9	9	12-week	Vibration
Adıguzel, et al. (2021)	Turkey	M/F	9	27.5	130	16.27	9	9	8-week	Pilates
Ameer, et al. (2019)	Egypt	M/F	6.2	34.16	115.15	25.83	10	10	/	Treadmill gait training
Chrysagis, et al. (2012)	Greece	M (*n* = 13) F (*n* = 9)	16.0	54.82	158.86	21.68	11	11	12-week	Treadmill training
Curtis, et al., (2017)	Denmark	M (*n* = 18) F (*n* = 10)	2–15	/	/	/	14	14	6-month	Segmental trunk and head control training
El-Basatiny, et al., (2014)	Egypt	M (*n* = 16) F (*n* = 14)	12.2	39.08	138.14	20.48	15	15	12-week	Backward walking training
El-Shamy, et al., (2014)	Egypt	M (*n* = 23) F (*n* = 7)	9.8	32.33	134	18	15	15	12-week	Whole-body vibration
Gatica-Rojas, et al., (2017)	Chile	M (*n* = 19) F (*n* = 13)	10.7	40.1	138	20.05	16	16	6-week	Wii-therapy
González, et al., (2020)	Spain	M (*n* = 15) F (*n* = 12)	12.5	/	/	20.35	14	13	6-week	Slackline training
Grecco, et al. (2013)	Brazil	M (*n* = 9) F (*n* = 5)	6.4	22.95	114.25	17.35	7	7	7-week	Treadmill gait training
Grecco-Collange, et al., (2013)	Brazil	M (*n* = 15) F (*n* = 18)	6.4	22.95	114.25	17.35	16	17	7-week	Treadmill training
Hemachithra, et al., (2019)	India	M (*n* = 12) F (*n* = 12)	2.75	10.3	85.2	14.2	12	12	Post intervention	Horse riding
Jelsma, et al., (2012)	South Africa	M/F	11.36	/	/	/	14	14	3-week	The Nintendo Wii Fit
Kara, et al., (2019)	Turkey	M (*n* = 14) F (*n* = 16)	11.53	41.06	144.23	18.99	15	15	12-week	Functional progressive strength and power training
Kepenek-Varol, et al., (2021)	Turkey	M (*n* = 14) F (*n* = 16)	11	/	/	18.17	15	15	8-week	Inspiratory muscle and balance training
Peungsuwan, et al., (2017)	Thailand	M (*n* = 8) F (*n* = 7)	13.25	36.96	138.5	19.27	8	7	8-week	Combined exercise training
Salem, et al., (2009)	USA	M (*n* = 6) F (*n* = 4)	6.53	/	/	/	5	5	5-week	Task-oriented training
Saxena, et al., (2016)	India	M (*n* = 8) F (*n* = 6)	10.31	32.71	133.85	18.26	7	7	2-day	Balance training with computerbased feedback
Surana, et al., (2019)	USA	M (*n* = 10) F (*n* = 14)	5.45	/	/	/	12	12	9-week	Lower-extremity functional training
Tarakci, et al., (2016)	Turkey	M (*n* = 19) F (*n* = 11)	10.5	/	/	/	15	15	12-week	Nintendo Wii-Fit balance-based video games
Wallard, et al., (2017)	France	M/F	8.95	29.20	129.0	17.55	14	16	4-week	Robotic-assisted gait training
Wallard, et al., (2018)	France	M (*n* = 15) F (*n* = 15)	8.95	19.2	122.0	12.90	14	16	4-week	Robotic-assisted gait rehabilitation
Yazıcı, et al., (2019)	Turkey	M/F	8.5	28.84	133.29	15.93	12	12	12-week	Robotic gait training

### Risk of bias assessment

**Fig. 2:**
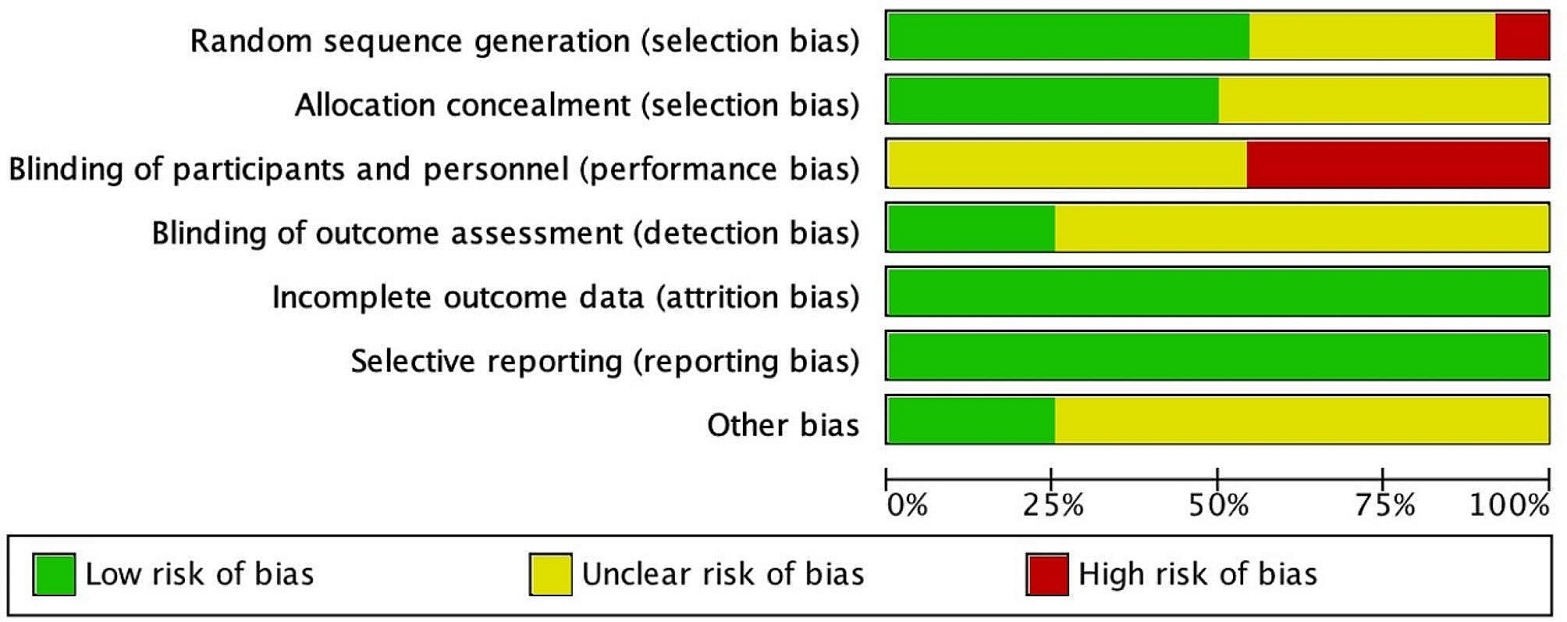
Risk of bias graph: review author’s judgements about each risk of bias item presented as percentages across all included studies

**Fig. 3:**
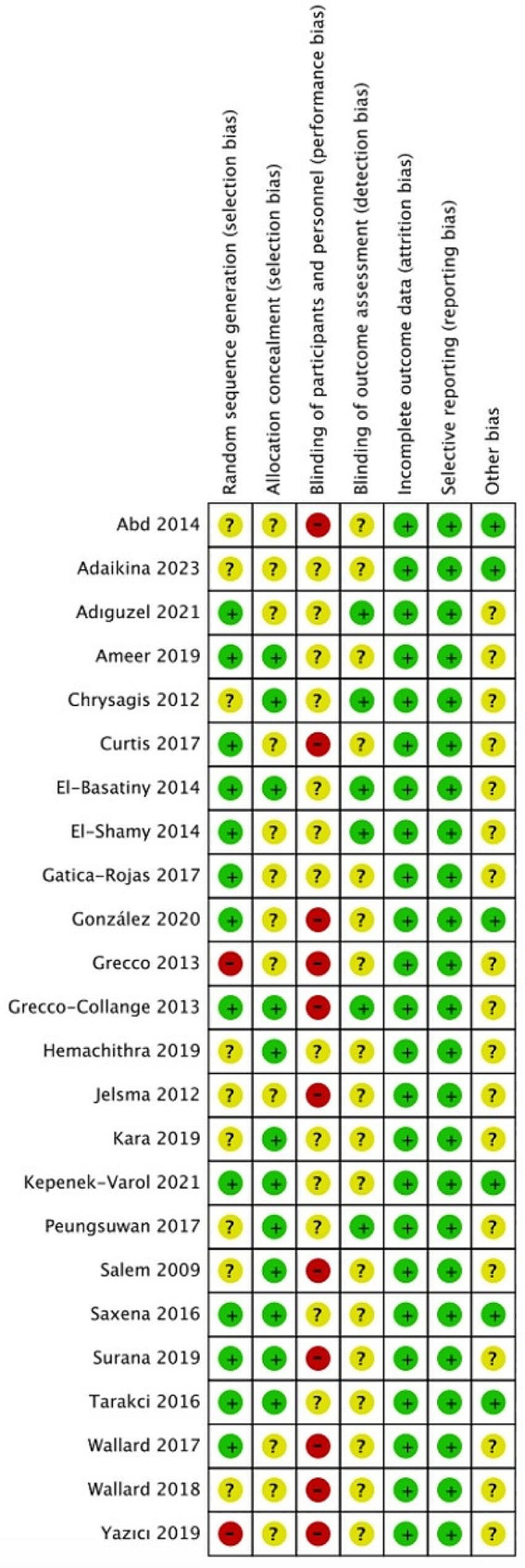
Risk of bias summary: review author’s judgements about each risk of bias item for each included study

The overview of the risk of bias in the included RCTs was assessed using the Cochrane Handbook version 5.1.0 tool in Figs. 2 and 3. And all included studies showed a low risk of bias in attrition bias and reporting bias. In Fig. 3, eleven studies are shown that present a high risk of bias in performance bias. The performance bias was significant due to the difficulty in masking subjects to group allocation during supervising training by therapists. Overall, the quality assessment indicated that all included studies had a low or moderate risk of bias.

### Meta-analysis

#### GMFM

**Fig. 4:**
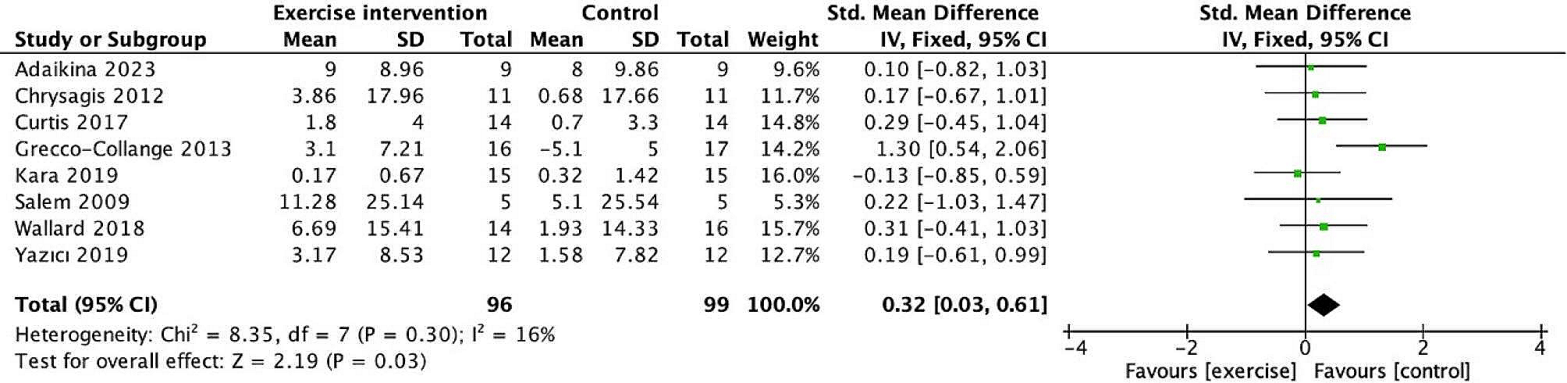
Forest plot of comparison: gross motor function measure (GMFM). 95% CI: 95% confidence interval; SD: standard deviation; IV: inverse variance

The meta-analysis in eight [33, 36, 37, 43, 46, 49, 53, 55] studies found a significant pooled effect of exercise on gross motor function compared to non-intervention or conventional therapy in children with cerebral palsy (SMD = 0.32, 95% CI 0.03 to 0.61, *p* = 0.03; heterogeneity: *I*^2^ = 16%; Chi^2^ = 8.35; *p* = 0.30) in Fig. 4.

### Gait speed

**Fig. 5:**
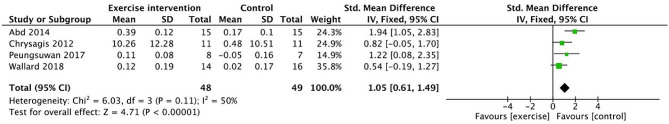
Forest plot of comparison: gait speed. 95% CI: 95% confidence interval; SD: standard deviation; IV: inverse variance

Figure 5 displays the inclusion of 97 children with cerebral palsy from four studies [32, 36, 48, 53]. The result indicated that gait speed was not improved with exercise compared to non-intervention or conventional therapy (SMD = 1.05, 95% CI 0.61 to 1.49, *p* < 0.00001), and there was significant heterogeneity (*I*^2^ = 50%, *p* = 0.11) in Fig. 5.

### Mobility

**Fig. 6:**
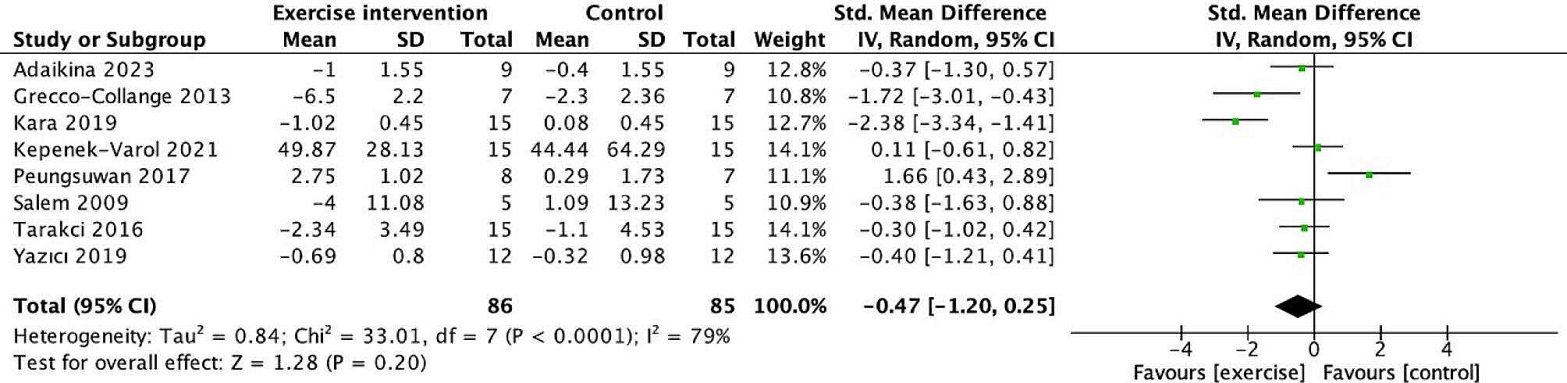
Forest plot of comparison: mobility. 95% CI: 95% confidence interval; SD: standard deviation; IV: inverse variance

Eight studies [33, 43, 46–49, 52, 55] conducted analyses to assess the impact of exercise on mobility in children with cerebral palsy in Fig. 6. The combined results of the eight trials demonstrated a significant increase in mobility in the exercise group compared to the control groups. The standardized mean difference was−0.47, with a 95% confidence interval ranging from−1.20 to 0.25. The p-value was 0.20, indicating an insignificant difference. There was a high level of heterogeneity (*I*^2^ = 79%), and the chi-square test yielded a value of 33.01 with a p-value of less than 0.0001.

### Muscle strength

**Fig. 7:**
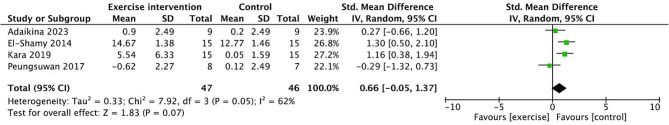
Forest plot of comparison: muscle strength. 95% CI: 95% confidence interval; SD: standard deviation; IV: inverse variance

The effect of exercise intervention on muscle strength was investigated and is shown in Fig. 7. The pooled analysis showed that exercise could not significantly improve muscle strength (SMD = 0.66, 95% CI−0.05 to 1.37, *p* = 0.07; heterogeneity: *I*^2^ = 62%; Chi^2^ = 7.92; *p* = 0.05), compared to the control group.

### Postural balance

**Fig. 8:**
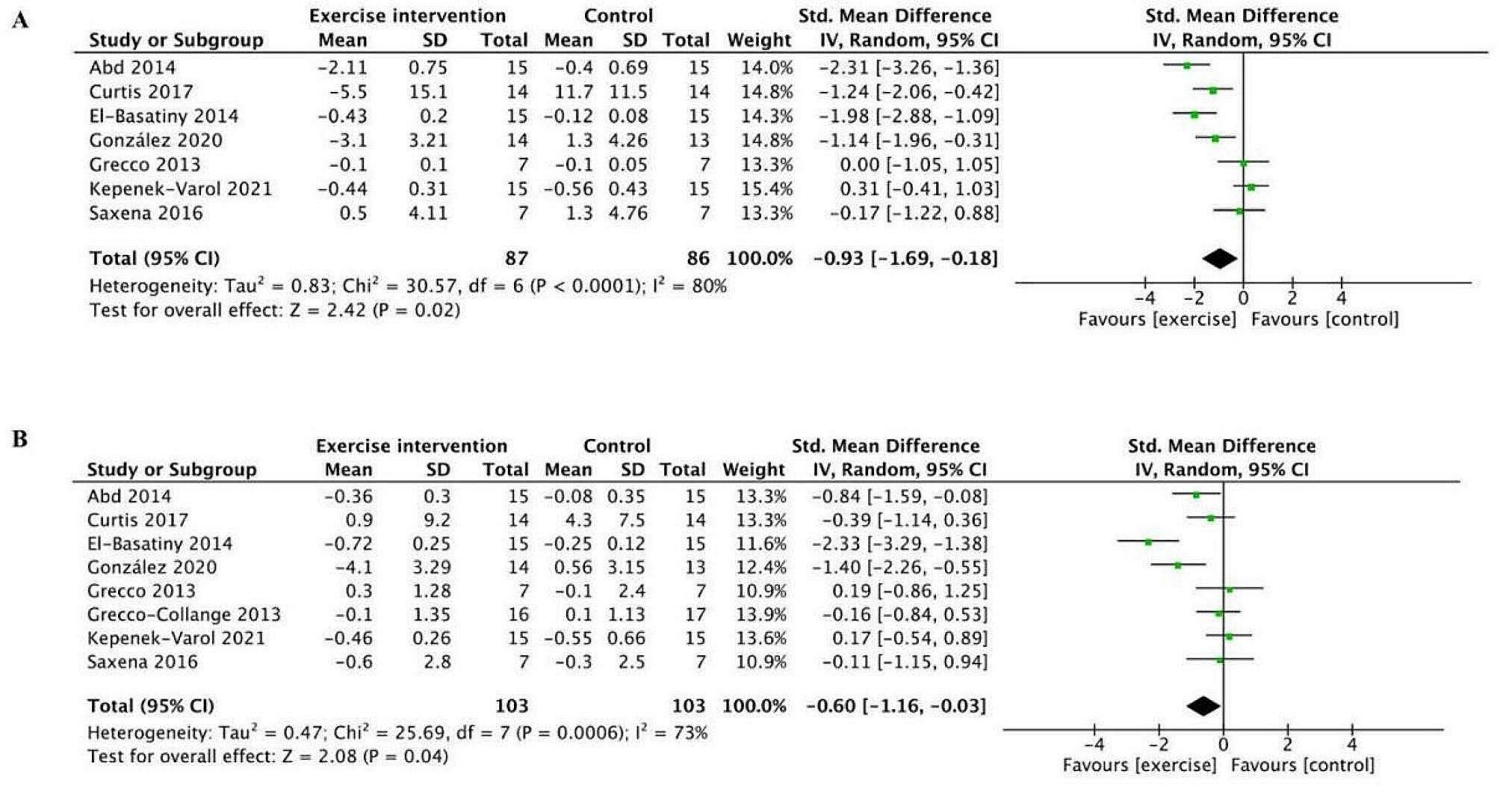
Forest plot of comparison: anteroposterior (A/P) stability index (**A**), and mediolateral (M/L) stability index (**B**). 95% CI: 95% confidence interval; SD: standard deviation; IV: inverse variance

Anteroposterior and mediolateral stability index were assessed, and the forest plot were depicted in Fig. 8A and B. Figure 8A demonstrates that the exercise had a substantial impact (Z = 2.42 (*p* = 0.02)) on improving balance in the anteroposterior (A/P) stability index. Similarly, the mediolateral (M/L) stability index exhibited a substantial alteration (Fig. 8B) (Z = 2.08 (*p* = 0.04)). There was a high level of heterogeneity (*I*^2^ = 80% and 73%), and the chi-square test yielded a value of 30.57 and 25.69 with a p-value of less than 0.0001 and 0.0006, respectively.

### Publication bias and sensitivity analyses

Funnel plots for GMFM, gait speed, mobility, muscle strength, A/P SI, and M/L SI are shown in supplementary Fig. 1. Sensitivity analysis of gait speed, mobility, muscle strength, A/P SI, and M/L SI indicated that our results were not significantly influenced by any singular study in supplementary Fig. 3. Begg’s test and Egger’s test showed no evidence of publication bias in GMFM (*p* = 0.902; *p* = 0.115), gait speed (*p* = 1.000; *p* = 0.507), mobility (*p* = 0.902; *p* = 0.718), muscle strength (*p* = 1.000; *p* = 0.999), A/P SI (*p* = 0.548; *p* = 0.133), and M/L SI (*p* = 0.386; *p* = 0.765) in supplementary Fig. 2.

## Discussion

This meta-analysis of RCTs assessed the efficacy of exercise interventions on postural balance, gait parameters, and muscle strength in children with CP. This systematic review and meta-analysis included 24 studies involving 579 children (age range: 2.5–16.0 years) with CP (Exercise intervention, *n* = 294; Control, *n* = 285). This meta-analysis revealed that exercise interventions are not associated with improved gait speed and muscle strength in children with cerebral palsy but with increased balance function and gross motor function and balance.

Several systematic reviews and meta-analyses have previously been conducted to investigate the efficacy of exercise interventions for patients diagnosed with CP. Evaluating gross motor function is crucial in a rehabilitation program for individuals with CP. In our meta-analysis, there was an improvement of GMFM by exercise intervention (*p* = 0.03), involving eight studies. In a review by Saquetto et al., there is also a significant improvement in GMFM E (MD = 2.97, 95% CI 0.07 to 5.86, *p* = 0.04), but no effect in GMFM (MD = 6.34, 95% CI−1.37 to 14.06, *p* = 0.11) by whole-body vibration, involving only two studies [56]. Enhancing a child’s gait is often the primary therapeutic objective for those with developmental disabilities [57]. There was no significant improvement in gait speed in our meta-analysis and no significant improvement in the other two studies of gait training [27] (MD = 0.92, 95% CI 0.19 to 1.66, *p* = 0.01) and whole-body vibration [56] (MD = 0.13, 95% CI 0.05 to 0.2, *p* = 0.0008). Balance is a crucial determinant of one’s ability to walk independently. In our study, the anteroposterior stability index and the mediolateral stability index are direct indicators used to evaluate the function of postural stability. Exercise intervention (A/P SI: *p* = 0.02; M/L SI: *p* = 0.04) and VR intervention [58] (SMD = 0.47, 95% CI 0.28 to 0.66, *p* < 0.00001) all have positive effects for children with CP. In a similar meta-analysis of exercise interventions for children with CP, exercise interventions (27 trials, including 834 children with CP) had no significant effect on gross motor function and were associated with higher levels of gait speed and muscle strength [25]. The potential reason for this result could be a search strategy about balance function in our mate-analysis, and further RCTs are needed to verify this result.

The beneficial effects of exercise interventions for children with CP may be associated with several possible mechanisms. CP causes a loss of muscular strength and bodily function by injury to the central nervous system, and muscle is a very pliable tissue that is also severely affected [59]. Resistance training, treadmill training, or combination training all involved some form of resistance training that aimed to improve muscle strength, power, or length deficits [60]. Resistance training interventions in children with CP can lead to muscle hypertrophy [61], increase muscle fiber diameter and muscle fiber length following the greater the number of actin-myosin interactions to contribute its strength [62]. Exercise can significantly enhance bodily balance by increasing the interplay between the neurological system and the muscles [63]. This collective endeavor encompasses interconnected systems, including the brain, neurons, and muscles [64]. Aerobic exercises, such as jogging and swimming, can stimulate neuronal activity, while strength training, such as weightlifting and using supports, can improve muscle coordination [65].

Among the 24 included studies, mostparticipants are above ten years old. Thus, early intervention is critical in maximizing long-term functionality in children with CP [20]. In addition, CP can lead to further complications, including limb stiffness, muscle strength weakness, muscular atrophy, skeletal abnormalities, and developmental coordination difficulties [18]. Balance training, treadmill training, whole-body vibration, Wii-Fit balance training, and robotic-assisted gait training are commonly related to developing balance function [30, 34, 37, 50, 51]. More importantly, exercise dosage, safety, and nervous system development should be focused on exercise intervention for children with CP.

### Strengths and limitations

This is the first systematic review and meta-analysis to examine the efficacy of exercise intervention with comprehensive related outcomes in children with CP. We expanded the associated outcomes, including GMFM, muscle strength, mobility, gait speed, A/P SI, and M/L SI. Furthermore, exercise may serve as the clinical practice guideline for children with CP. This study has some limitations that should be noted: (1) We did not determine the specific exercise type for children with CP. (2) Due to the partition difficulty of variables about age, duration, and exercise type, we did not report subgroup analysis in this manuscript. Further recommendations on the optimal exercise protocol were limited. (3) Increased heterogeneity may also be caused by factors that are not assessed or reported measurement mistakes and poor research plan execution. (4) We only used three databases and did not search other databases (e.g., CINAHL, PEDro, etc.), which needed more comprehensiveness of the search. Future research should concentrate on rigorous RCTs, standardizing measuring methods, expanding the number of participants, and improving experimental design to decrease heterogeneity and increase the valid evidence.

## Conclusions

The present systematic review and meta-analysis present evidence that exercise significantly improves gross motor function and balance in children with cerebral palsy. This review enhances the significance and relevance of exercise, especially in balance for children with cerebral palsy. These findings can assist clinicians in formulating exercise prescriptions more effectively. Further, it can offer researchers valuable insights into the study of children with cerebral palsy to explore additional aspects such as duration of exercise, safety, motion parameters, and balance function.

### Electronic supplementary material

Below is the link to the electronic supplementary material.

Supplementary Material 1

## Data Availability

All data generated or analyzed during this study are included in this published article and its Supplemental Digital Content. The datasets generated during and/or analyzed during the current study are publicly available.

## Abbreviations

CP: Cerebral palsy
RCTs: Randomized controlled trials
GMFCS: Gross motor function classification system
PRISMA: Preferred reporting items for systematic reviews and meta-analyses
SMD: Standard mean differences
SD: Standard deviation
GMFM: Gross motor function measure
A/P: Anteroposterior
L/M: Mediolateral
