# Supplementary material for: Effects of exercise intervention on balance function in children with cerebral palsy: a systematic review and meta-analysis of randomized controlled trials
Source: BMC Sports Sci Med Rehabil. 2024 Aug 7;16:164. doi: 10.1186/s13102-024-00922-5 (PMC11305018; doi:10.1186/s13102-024-00922-5)
Supplement: Supplementary file 1 — Supplementary Material 1 [file 13102_2024_922_MOESM1_ESM.docx]

Supplementary Material

# Supplementary Figures

**Online supplementary figure 1.** Funnel plots of (A) GMFM, (B) gait speed, (C) mobility, (D) muscle strength, (E) A/P SI, and (F) M/L SI.

**
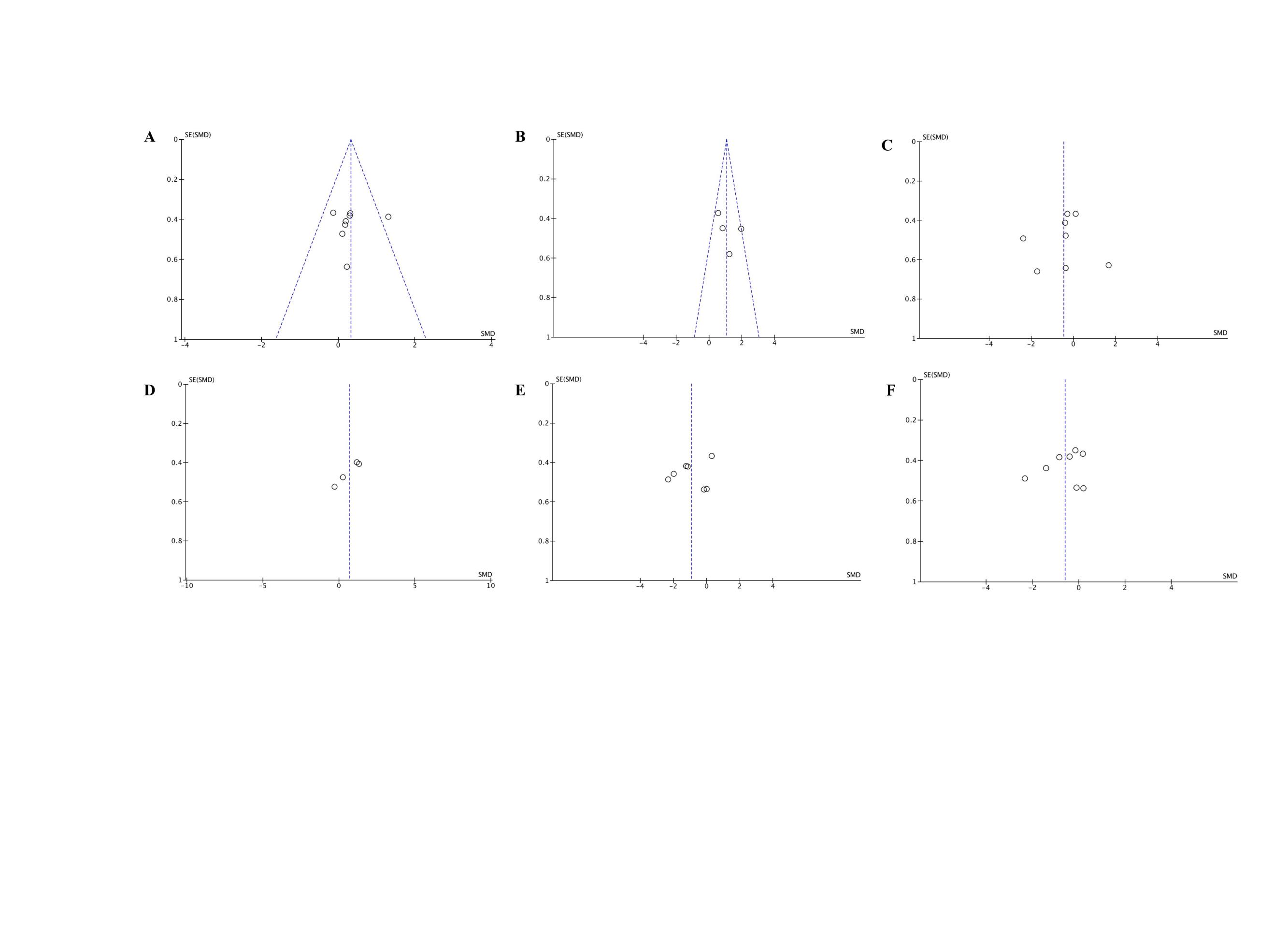
**

**Online supplementary figure 2.** Begg’s funnel plots of (A) GMFM, (B) gait speed, (C) mobility, (D) muscle strength, (E) A/P SI, and (F) M/L SI.

**
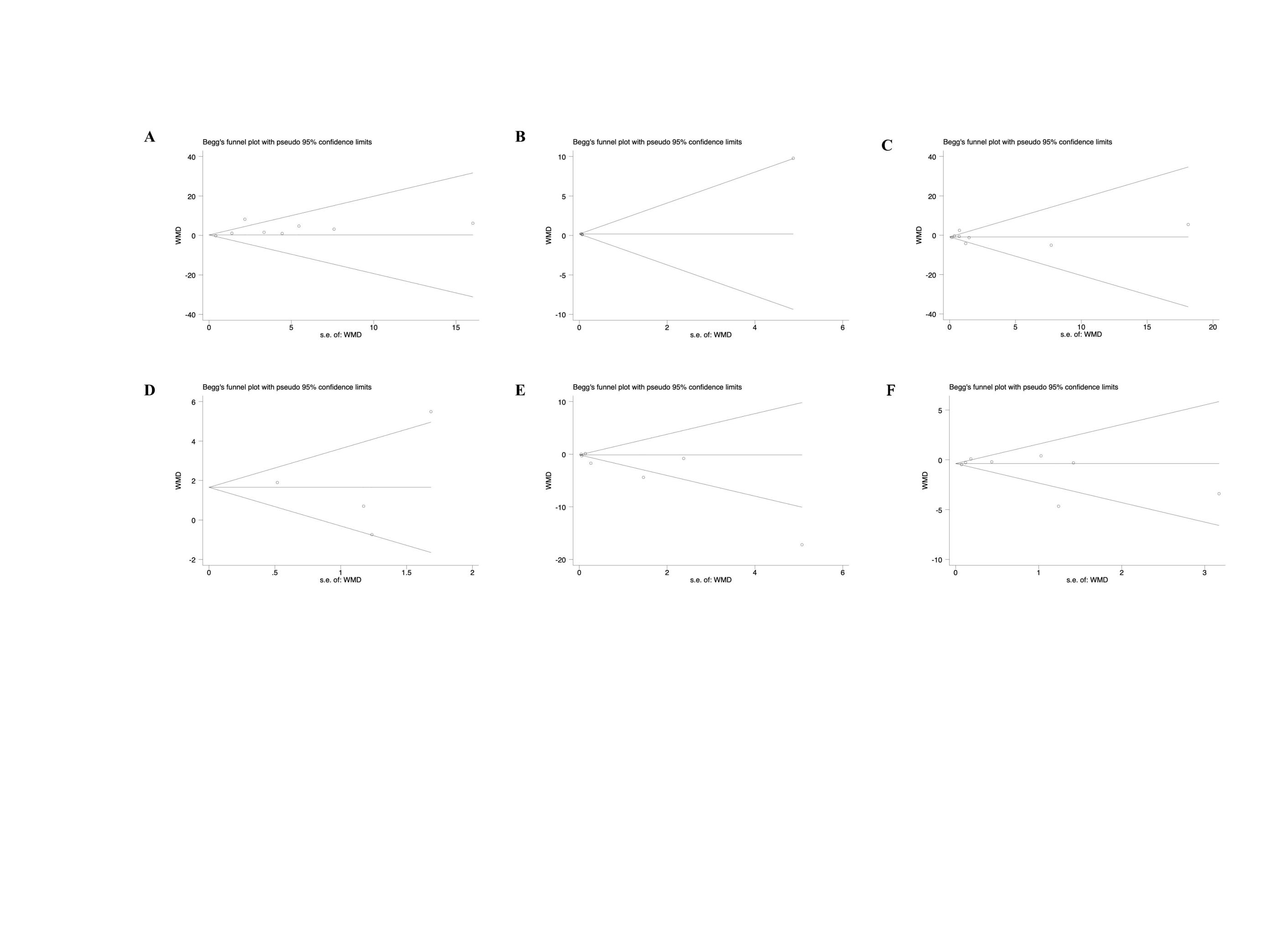
**

**Online supplementary figure 3.** Sensitivity analysis of (A) GMFM, (B) gait speed, (C) mobility, (D) muscle strength, (E) A/P SI, and (F) M/L SI.

**
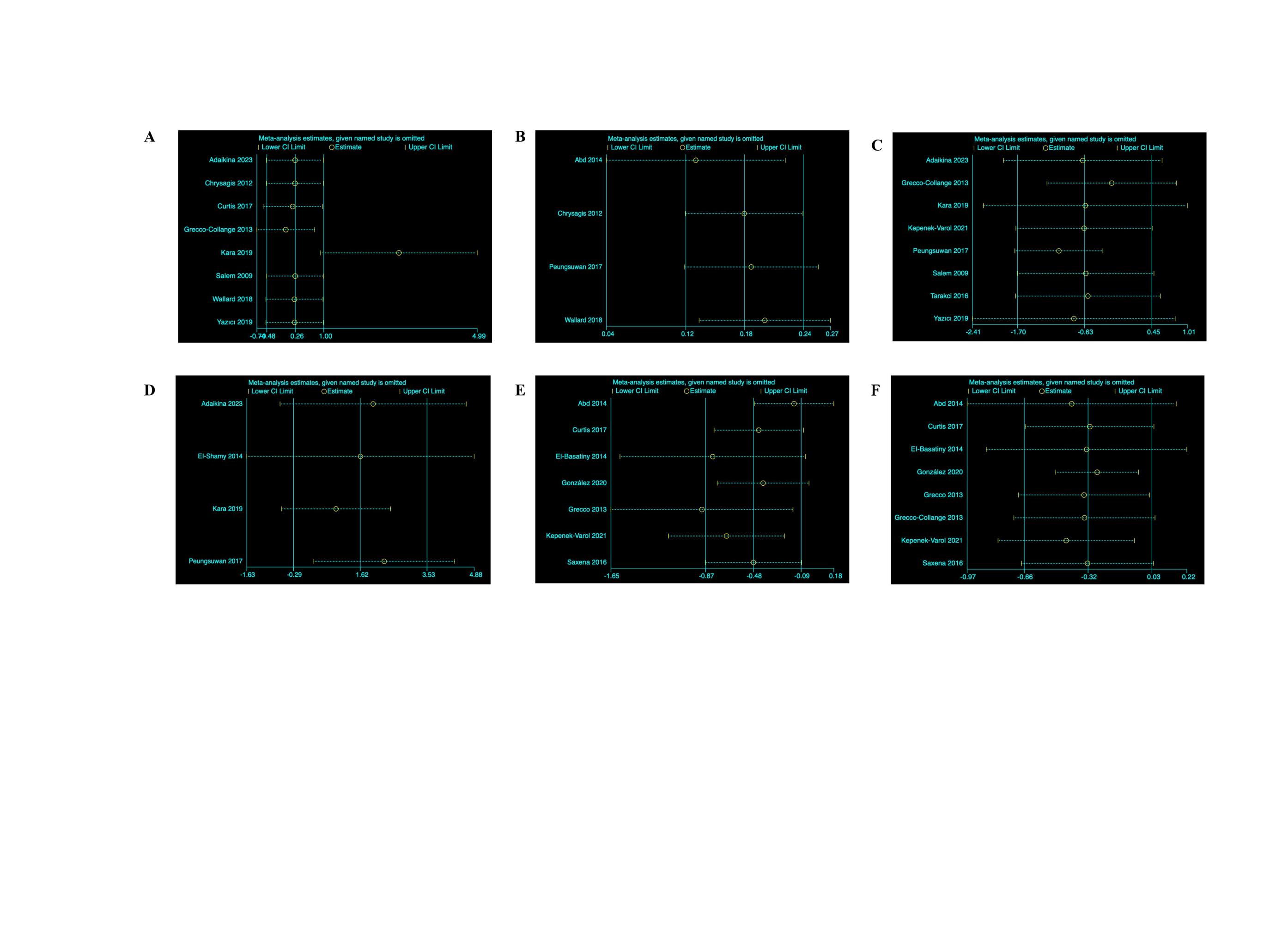
**

**Online supplementary figure 4.** Forest plot of comparison: stability index. 95% CI: 95% confidence interval; SD: standard deviation; IV: inverse variance.


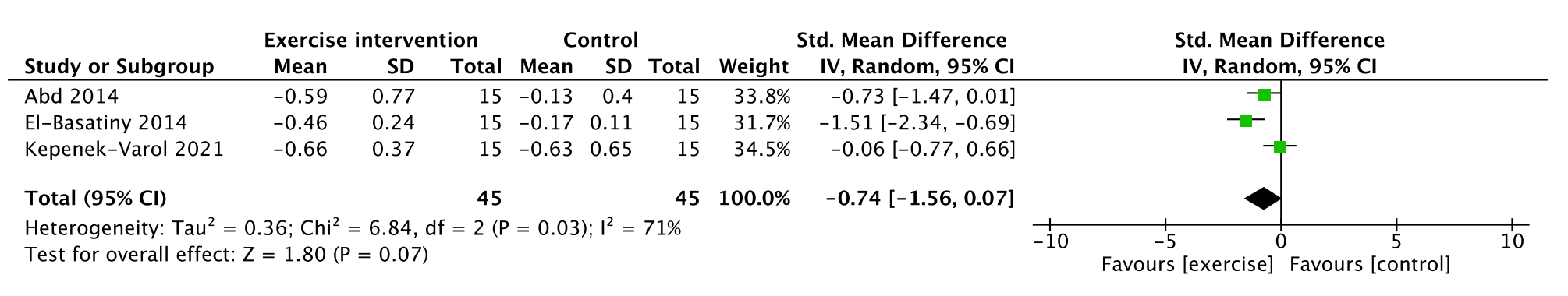


**Online supplementary figure 5.** Forest plot of comparison: step length. 95% CI: 95% confidence interval; SD: standard deviation; IV: inverse variance.


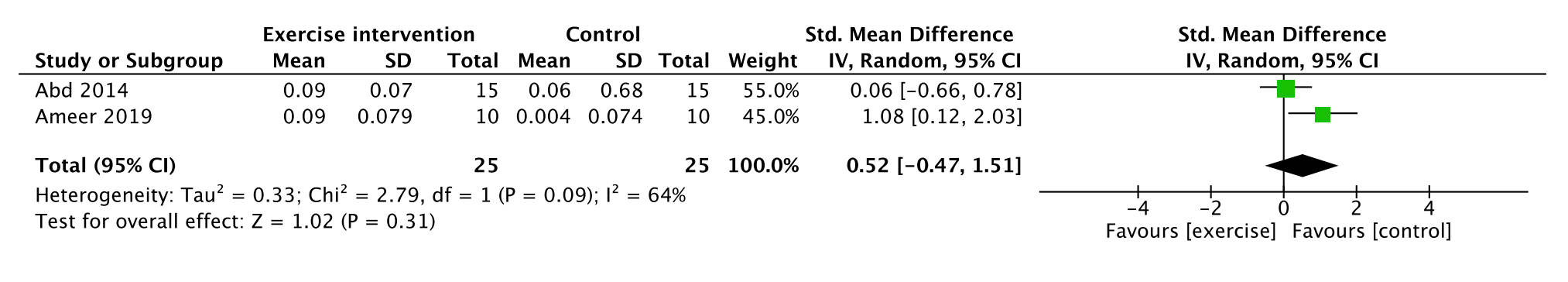


# Supplementary Tables

**Online supplementary table 1.** Search syntax.

| ***Database*** | ***Search strategy*** | ***Result*** |
| --- | --- | --- |
| Pubmed  N=304 | ("cerebral palsy"[MeSH Terms] OR ("cerebral"[All Fields] AND "palsy"[All Fields]) OR "cerebral palsy"[All Fields]) AND ("child"[MeSH Terms] OR "child"[All Fields] OR "children"[All Fields] OR "child s"[All Fields] OR "children s"[All Fields] OR "childrens"[All Fields] OR "childs"[All Fields]) AND ("exercise"[MeSH Terms] OR "exercise"[All Fields] OR "exercises"[All Fields] OR "exercise therapy"[MeSH Terms] OR ("exercise"[All Fields] AND "therapy"[All Fields]) OR "exercise therapy"[All Fields] OR "exercising"[All Fields] OR "exercise s"[All Fields] OR "exercised"[All Fields] OR "exerciser"[All Fields] OR "exercisers"[All Fields]) AND ("balance"[All Fields] OR "balanced"[All Fields] OR "balances"[All Fields] OR "balancing"[All Fields]) | 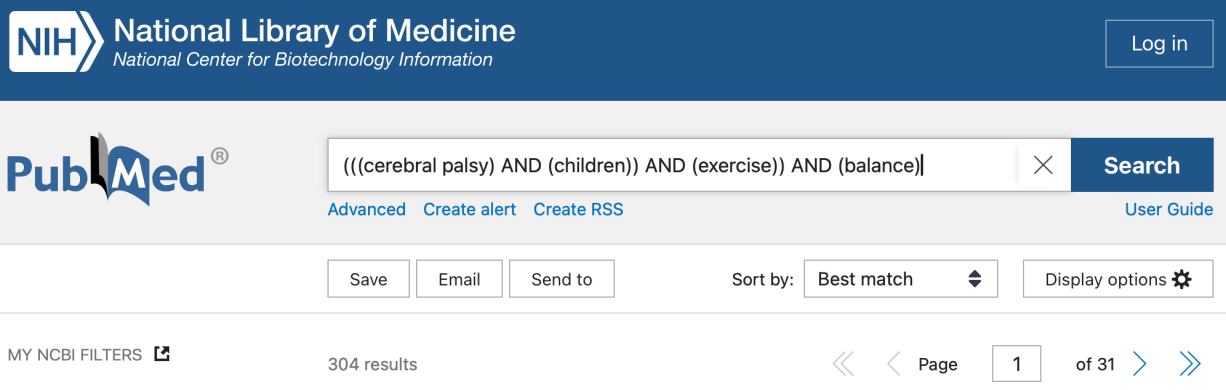 |
| Web of Science  N=293 | cerebral palsy (All Fields) and children (All Fields) and exercise (All Fields) and balance (All Fields) | 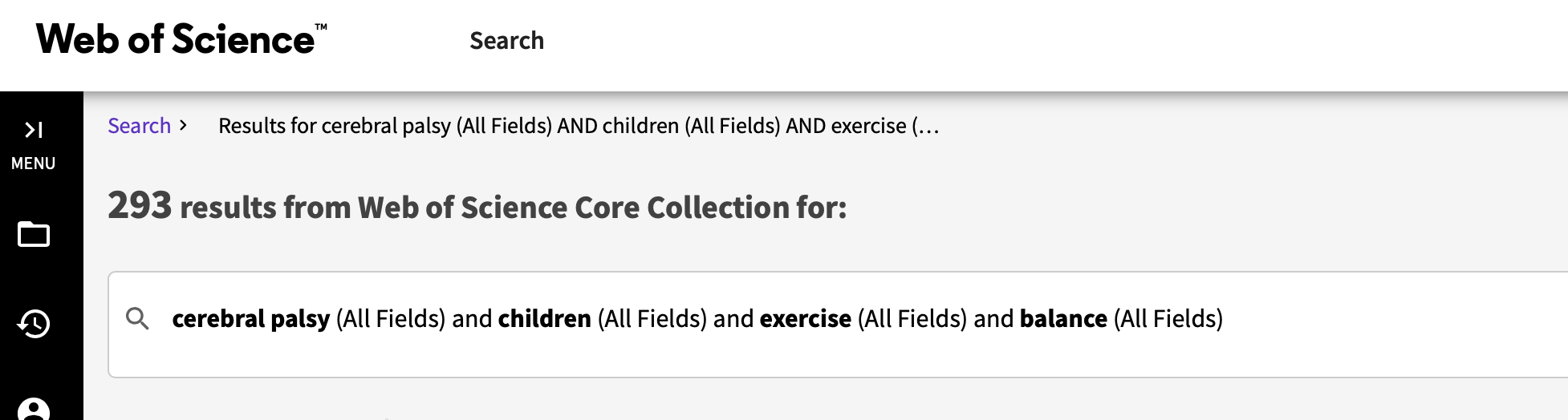 |
| Scopus  N=290 | ( TITLE-ABS-KEY ( cerebral AND palsy ) AND TITLE-ABS-KEY ( children ) AND TITLE-ABS-KEY ( exercise ) AND TITLE-ABS-KEY ( balance ) ) | 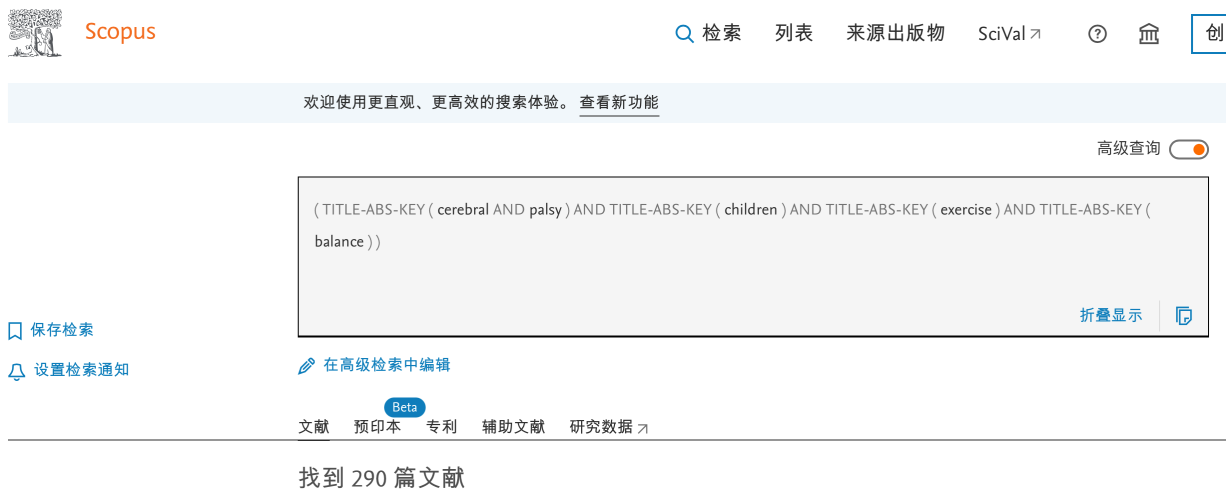 |

**Online supplementary table 2.** Intervention protocol of included studies.

| ***Study*** | ***Cerebral palsy classification*** | | ***Intervention protocol*** |
| --- | --- | --- | --- |
|  | ***Spasticity grades*** | ***GMFCS level*** |  |
| Abd, et al. (2014) | 1: n=9 (30%)  1+: n=16 (53%)  2: n=5 (17%) | I: n=13 (43%)  II: n=17 (57%) | 30 mins of dynamic postural stability training, stability level 8 for 4 weeks followed by training on stability level 7 during the next 4 weeks |
| Adaikina, et al. (2023) | Spastic: n=7 (78%)  Dyskinetic: n=2 (22%) | I: n=3 (33%)  II: n=5 (66%)  III: n=1 (1%) | Home-based VT sessions (standing barefoot on the plate four times a week, 9 minutes a day, at a frequency of 20 Hz and amplitude of 1.5–2.0 mm) |
| Adıguzel, et al. (2021) | / | I: n=6 (33.3%)  II: n=11 (61.1%)  III: n=1 (5.6%) | 2 days a week, 1 h a day, for 8 weeks in modifed pilates exercises |
| Ameer, et al. (2019) | Mild spasticity | / | Treadmill gait training for 20 minutes at a self-paced speed |
| Chrysagis, et al. (2012) | / | 1. II: n=14 (64%)   III: n=8 (36%) | Participants walked on the treadmill without body weight support to a comfortable speed for a maximum of 30 mins per session at a frequency of three times per week |
| Chrysagis, et al. (2012) | Spastic: n=16 (57%)  Dyskinetic: n=12 (43%) | III: 5 (18%)  IV: 6 (21%)  V: 17 (61%) | Trained head and trunk control for up to a half hour, 5 days a week for a period of 6 months by a published algorithm |
| El-Basatiny, et al., (2014) | 1: n=8 (27%)  1+: n=17 (57%)  2: n=5 (16%) | I: n=14 (47%)  II: n=16 (53%) | Walking training 25 min/day, 3 days/week for 3 successive months |
| El-Shamy, et al., (2014) | / | I: n=13 (43%)  II: n=17 (57%) | Using the Vibraflex Home Edition II WBV device;  3 mins of WBV, 3 mins of rest, 3 mins of WBV, 3 mins of rest, and 3 mins of WBV;  12 Hz vibration frequency in the first treatment sessions, and increase the WBV frequency in steps of 0.5 Hz every two treatment sessions until the target frequency of 18 Hz |
| Gatica-Rojas, et al., (2017) | / | I: n=6 (19%)  II: n=11 (34%) | Included stretching, flexibility, strengthening, and balance exercises for 40 minutes in each session at a frequency of three times per week over 6 weeks |
| González, et al., (2020) | / | I: n=16 (59%)  II: n=11 (41%) | Included a 10-min warm-up period with stretching of the main locomotive system muscles, followed by 15 min of slackline tasks, and ending with a 5-min cool-down phase including active and static stretching, three 30-min sessions per week |
| Grecco, et al. (2013) | / | I: n=7 (50%)  II: n=3 (21%)  III: n=4 (29%) | Walked at 60% maximal speed in the first and final five minutes of the session and walked at 80% maximal speed for the other 20 minutes, two 30-minute sessions per week |
| Grecco-Collange, et al. (2013) | / | I: n=13 (39%)  II: n=15 (45%)  III: n=5 (15%) | Walked at 60% maximal speed in the first and final five minutes of the session and walked at 80% maximal speed during the other 20 minutes, two 30-minute sessions per week over seven  consecutive weeks |
| Hemachithra, et al., (2019) | / | / | 30 min of the horse riding simulator U-Gallop |
| Jelsma, et al., (2012) | / | / | Stood on the Wii Fit balance board operating the gaming for 3-week |
| Kara, et al., (2019) | / | I: n=14 (47%)  II: n=9 (30%)  III: n=7 (23%) | Consisted of functional strengthening using the leg press (for eccentric, concentric, and isometric contraction of quadriceps femoris, hamstrings, tibialis anterior, and gastrosoleus), plyometric exercises (including jumping), and balance training, treated 3 times per week for 90 minutes per session for a total of 36 sessions over a period of 12 weeks |
| Kepenek-Varol, et al., (2021) | / | I: n=18 (60%)  II: n=12 (40%) | Including NDTbased balance and postural control exercises, and balance exercises with the BBS for 8 weeks with three training sessions of 45 minutes per week |
| Peungsuwan, et al., (2017) | / | I: n=4 (27%)  II: n=8 (53%)  III: n=3 (20%) | Consisted of a 5-minute warm-up period, 60 minutes of circuit exercises, and a 5-minute cool-down period for 8 weeks;  Functional strength training consisted of a sit-to-stand (STS) and a step up-down (SUD) activity;  Endurance training was performed the 3 stations for endurance training included (1) leg stationary bicycles, (2) elliptical machines, and (3) recreational fast walking or running |
| Salem, et al., (2009) | / | I: n=2 (20%)  II: n=6 (60%)  III: n=2 (20%) | (1) walking activities ; (2)walking up and down ramps and stairs; (3) stepping forward, backward, and sideways from floor onto blocks of various heights; (4) standing balance activities; (5) standing up from a chair; (6) performing single leg stance; and (7) kicking a ball, twice weekly for 5 weeks |
| Saxena, et al., (2016) | / | II: n=6 (43%)  III: n=8 (57%) | Stood on the force plate of the Good balance system for 15 min and two such sessions in a day for two days |
| Surana, et al., (2019) | / | I: n=8 (33%)  II: n=16 (67%) | (1) motor learning, (2) intensive and structured practice, (3) skill or activity progression, and (4) resistance training, 90 hours for 9 weeks |
| Tarakci, et al., (2016) | Hemiplegic: n=14 (47%)  Diplegic: n=12 (40%)  Dyskinetic: n=4 (13%) | / | Consisted of two days a week (1 sessions - 50 minutes) with total of 12 weeks; Wii-Fit balance-based video games group were applied to Wii Group (WiiG) for 20 minutes |
| Wallard, et al., (2017) | / | / | Received twenty sessions of Lokomat®Pediatric conducted over a four weeks period (5 sessions/week), gait speed was initially set at 0.7 km/h and gradually increased to 1.4 km/h |
| Wallard, et al., (2018) | / | / | consisted of twenty Lokomat® Pediatric sessions with a duration of 40 min, spread over a period of four weeks, the initial body-weight support was 70%, and was then gradually decreased to 40% over the sessions |
| Yazıcı, et al., (2019) | / | / | During aerobic exercises, a low-intense 5-min warm-up and cooling program was performed at 30-40% of the maximum HR;  brisk walking was performed for 20 min with increasing speed at 55-75% of the maximum HR;  Gait training comprised a 30-min active walking training |

**Online supplementary table 3.** Outcomes of included RCTs (Experimental/Control).

| Author | GMFM | gait speed | Mobility | Muscle strength | Step length | Overall SI | A/P SI | M/L SI |
| --- | --- | --- | --- | --- | --- | --- | --- | --- |
| Abd, et al. (2014) | / | 0.39±0.12 (m/s) | / | / | 0.09±0.07 (cm) | -0.59±0.77 | -2.11±0.75 | -0.36±0.30 |
|  | / | 0.17±0.10 | / | / | 0.06±0.68 | -0.13±0.40 | -0.4±0.69 | -0.08±0.35 |
| Adaikina, et al. (2023) | 9±8.96 | / | -1.0±1.55 (s) | 0.9±2.49 (kg) | / | / | / | / |
|  | 8±9.86 | / | -0.4±1.55 | 0.2±2.49 | / | / | / | / |
| Ameer, et al. (2019) | / | / | / | / | 0.09±0.079 (m) | / | / | / |
|  | / | / | / | / | 0.004±0.074 | / | / | / |
| Chrysagis, et al. (2012) | 3.86±17.96 | 10.26±12.28  (m/min) | / | / | / | / | / | / |
|  | 0.68±17.66 | 0.48±10.51 | / | / | / | / | / | / |
| Curtis, et al., (2017) | 1.8±4.0 | / | / | / | / | / | -5.5±15.1 (mm) | 0.9±9.2 (mm) |
|  | 0.7±3.3 | / | / | / | / | / | 11.7±11.5 | 4.3±7.5 |
| El-Basatiny, et al., (2014) | / | / | / | / | / | -0.46±0.24 | -0.43±0.20 | -0.72±0.25 |
|  | / | / | / | / | / | -0.17±0.11 | -0.12±0.08 | -0.25±0.12 |
| El-Shamy, et al., (2014) | / | / | / | 14.67±1.38 (N·m) | / | / | / | / |
|  | / | / | / | 12.77±1.46 | / | / | / | / |
| González, et al., (2020) | / | / | / |  | / | / | -3.1±3.21 (mm/s) | -4.1±3.29  (mm/s) |
|  | / | / | / | / | / | / | 1.3±4.26 | 0.56±3.15 |
| Grecco, et al. (2013) | / | / | / | / | / | / | -0.1±0.10 (cm) | 0.3±1.28 (cm) |
|  | / | / | / | / | / | / | -0.1±0.05 | -0.1±2.40 |
| Grecco-Collange, et al. (2013) | 3.1±7.21 | / | -6.5±2.2 (s) |  | / | / | -0.4±0.0 | -0.1±1.35 |
|  | -5.1±5.0 | / | -2.3±2.36 |  | / | / | -0.1±0.0 | 0.1±1.13 |
| Kara, et al., (2019) | 0.17±0.67 | / | -1.02±0.45 (s) | 5.54±6.33 (N/kg) | / | / | / | / |
|  | 0.32±1.42 | / | 0.08±0.45 | 0.05±1.59 | / | / | / | / |
| Kepenek-Varol, et al., (2021) | / | / | 49.87±28.13 (m) |  |  | -0.66±0.37 | -0.44±0.31 | -0.46±0.26 |
|  | / | / | 44.44±64.29 |  |  | -0.63±0.65 | -0.56±0.43 | -0.55±0.66 |
| Peungsuwan, et al., (2017) | / | 0.11±0.08 (m/s) | 2.75±1.02 (s) | -0.62±2.27 (rep) |  | / | / | / |
|  | / | -0.05±0.16 | 0.29±1.73 | 0.12±2.49 |  | / | / | / |
| Salem, et al., (2009) | 11.28±25.14 | / | -4.00±11.08 | / | / | / | / | / |
|  | 5.10±25.54 | / | 1.80±13.23 | / | / | / | / | / |
| Saxena, et al., (2016) | / | / | / | / | / | / | 0.5±4.11 | -0.6±2.8 |
|  | / | / | / | / | / | / | 1.3±4.76 | -0.3±2.5 |
| Tarakci, et al., (2016) | / | / | -2.34±3.49 | / | / | / | / | / |
|  | / | / | -1.1±4.53 | / | / | / | / | / |
| Wallard, et al., (2018) | 6.69±15.41 | 0.12±0.19 | / | / | / | / | / | / |
|  | 1.93±14.33 | 0.02±0.17 | / | / | / | / | / | / |
| Yazıcı, et al., (2019) | 3.17±8.53 | / | -0.69±0.80 | / | / | / | / | / |
|  | 1.58±7.82 | / | -0.32±0.98 | / | / | / | / | / |

Abbreviations: GMFM (gross motor function measure); PBS (the pediatric balance test); TUG (time up and go); SI (stability index); A/P SI (anteroposterior stability index); M/L SI (mediolateral stability index).
